# Editorial

**Published:** 1871-06

**Authors:** 


### Editorial.

### *Parke, Jennings & Co.*

Our readers, have, of course, read the advertisement of this firm on the cover of the JOURNAL. The advertising and editorial pages of this Journal are entirely distinct. The publishers control the former, the editors the latter. But simple justice to a highly deserving and enterprising firm requires that we should say that they are doing a great work in the Northwest. From careful trial of many of their preparations, in our private practice, we are prepared to say that they have surprised us by a purity and perfection which we have rarely seen equaled.

Not as a compliment to the firm, but as a personal guarantee from personal experience, we are prepared to say, that Messrs. Parke, Jennings & Co. have succeeded in furnishing the profession medicines which are prepared with the most exquisite care, and may be depended upon for producing their typical effects. We can most cordially recommend their various preparations to our friends and the profession generally.

It will be noticed that the long-time agent of Tilden & Co., Mr. E. BENNETT, has become associated with Messrs. Parke, Jennings & Co., and his familiar face and well known thorough acquaintance with the drug business, in all its various ramifications, will inspire appropriate confidence in all those who are aware of his conscientious devotion to the business of introduction to the profession of absolutely pure and reliable medicines. We have known Mr. Bennett for many years, and firmly believe that he will never knowingly advocate or seek to introduce other than the very best of drugs and chemicals.

This notice, we repeat, is no mere puff, but given on the personal responsibility of the senior editor as eminently due a highly deserving enterprise, and several wholly reliable gentlemen.

### *Half-Yearly Compendium.*

No. VII of this valuable American syllabus of valuable articles from the medical press of this country has come to hand. $3. per annum; single Nos. $2; Nos. 1 to 6 inclusive, $7. Address S. W. Butler, M.D., 115 S. Seventh street, Philadelphia. The publisher sends us the following card:

AN EXPLANATION.

The very late appearance of this number of the Half-Yearly Compendium has been caused by the loss of a large and important portion of copy by a printer. The fact was concealed from us for a long time, while frivolous excuses were given for the delay, and when we were apprised of the loss, considerable time was required to supply new copy.

To us this unfortunate delay has been a positive, downright loss, and the damage can scarcely be estimated by dollars and cents. To our subscribers it has been an annoyance the cause of which could not well be explained in detail, and we have sufficient confidence in them to believe that this explanation will be accepted, and that they will forgive and forget.

S. W. BUTLER.

### *American Journal of Obstetrics.*

Wm. Baldwin & Co., 21 Park Row, New York, have assumed the publication of this now standard Journal. Issued quarterly, at $5 a year. Single copies, $1.50. It is unnecessary for us to repeat that it is the best periodical extant on the subjects to which it is devoted. The names of Dawson, Nœggerath and Jacobi, the editors, alone, are a sufficient guarantee of its practical worth and excellence. Don't be satisfied merely with Prof. ——'s superficial twaddle on the woman question, but read, mark, learn and inwardly digest what those who know, have to say and write about it.

### *Lying Made Easy.*

The inventive genius of the American mind has been credited already with much that is ingenious, much that is useful, and much that is detrimental to humanity. Among the first two divisions, as eminently typical of both, we would classify the art of printing; among the second two, as likewise representing both, might be classed the manufacture of lucifer matches, and nitro-glycerine. Under the last head, as peculiarly representative thereof, should be ranged the art of lying, as applied to the manufacture of "medical cases" for publication. In looking over the published lists of patents issued to Western inventors we regret to observe the absence of a notice of the issue of letters patent for this manufacture, because we hope it will be made the strict monopoly of its present holders and carefully protected from infringement, in order that the professional public may always be able to judge the quality of the article by the "trade mark," the maker's name, without being subjected to the necessity of a critical personal explanation; and also, that, as in the case of the chemical above referred to, when the inevitable explosion occurs, the inventor, and he alone, may be "hoist with his own petard."

Within a comparatively short time, several cases of deliberate fraud have been brought to the knowledge of this Journal. Two of these we have taken occasion to notice in such a manner as to induce readers to criticise them carefully, and thus detect their true character. We have only done so after careful investigation and full proof, in the hope that the hint would be sufficient.

In the future, under similar circumstances, we shall, after having gathered all the facts, publish them in detail without fear or favor. W. H.

The disagreements of doctors have become proverbial; but while admitting their frequency, we must plead in palliation the sometimes vague and doubtful data upon which they are called upon to make diagnosis.

We submit to our readers the following verbatim copy of a letter, by the aid of which we were expected to "give an opinion" of a case. If any of our professional brethren can throw any light upon the subject, we promise to make all due allowance for honest differences of opinion. W. H.

"Dr doctor my litle boy 5 years and 8 mounths he has taking sick on the 6 of december when i notice hem geting sick he compleiand of every Joint in body geting sore then a few days after he got feeeble and then sweld up every Joint in his body even to the solls of his feet then he got stife

"he a vilent beating on he hart and is all the time sweeting and when he gose to sleep he moans and sys if you hard hem you would thenk that his hart would break he coff all the time and heef of throws of big flams he bely is swell and and hard he lips swelled up he is picking he nose and lip and fingears neals he eat nothing he compleans of a in he bely and in stomick and he sides he water after setling torns yallow and crooddles he is real scelliton this last 4 or 5 day back he complens of sumting resing up to he troatle and choaking hem he is needing very often and cannot pass nothing except when he get medcin so you are recommended to me on yesterday as i had trile of a good many eotors before you i thought try you"

[COMMUNICATED.]

KEELER, April 30, 1871.

J. ADAMS ALLEN, M.D.

*Sir*—Mrs. G., aged 32, died 28th; postmort. 30th, to-day; abdomen of large size distended to utmost capacity, tapped to draw off fluids, considerable gas escaped; made incision from ensiform cartilage to midway between umbilicus and pubes; on opening to the peritoneum found it white, firm and adherent to the morbid growth; dissecting the soft parts, removed about ten pounds, eight inches in width, 2½ thick in centre, and extending from side to side, saddle-shaped. We now removed about three pints fluid, yellowish brown lymph; think there were some pus globules in it; found the whole parietes lined with a substance from one to one and a half inches in thickness; in most places it could be broken up with the hand into a lumpy substance of a greenish color, looking exactly as though you had taken a handful of jelly, closed your hand upon it and broken it up. This substance was found everywhere except on the diaphragm and a strip about four inches wide along the spine extending down to the anus where the bowels were firmly pressed against the spine, there the peritoneum was smooth to the feel, and there appeared to be no deposit of this peculiar substance there; in places it was firmer from the umbilicus down to the bowels, it was firmer at the attachment to the parietes, and all the attachments to the bowels were easily broken up with the fingers, denuding the bowels of their peritoneal covering. In other respects the bowels were healthy. The liver was pressed up and to the right side, being bound by strong ligaments, too strong to be broken up by the fingers readily; the uterus was small; no enlargement of any organ, but from long sickness and strong pressure they were diminished in size.

I send you a sample of the most organized parts, some of it being quite firm; one portion from the large tumor near the umbilicus, involving the omentum, peritoneal covering of the bowels, and parietal peritoneum; the other portion taken from the broad ligament. There was one opening into the bowel, perhaps four lines, forming a cul-de-sac in the substance one and a quarter inches in depth, but saw no fecal matter, yet it could have passed into it from the bowel. The whole growth sprung from the lining membrane of the parietes of the abdomen as grass grows from the lawn. One and a half years since, I diagnosed the case chronic peritonitis, and have ever since held to the opinion. The substance had a feel like glue, and when dried on the fingers a little, would glue them together quite strongly; the whole amount of it would probably weigh thirty-five pounds.

Our examination was hurried and bungled through for want of time. I will be much obliged to you if you will examine the specimen and let me know what you think about it. The specimen sent you is of the most fibrous cellular specimen to be procured, having cut it from the most solid part, yet by handling them they have parted with the greater part of the gelatinous substance, leaving the fibrous portions more by themselves.

That such a large amount of substance should accumulate in two years, all from the peritonea membrane, is out of the common course of events, no other organs being involved except the small glands, being, as it were, swallowed up by the growth.

Yours, truly, GEO. BARTHOLOMEW.

EDS. NOTE. The specimen accompanying the above communication when subjected to microscopic examination presented the characteristic appearances of colloid cancer; consisting of cells presenting every stage of perversion from the typical cell of normal epithelium to the nucleated fibre cell; these extremes, however, being in the minority; the principal portion consisting of large irregularly ovoidal and fusiform multinucleated and nucleolated cells, with abundant free nuclei; the whole being evidently proliferated from the epithelial layer of the peritoneum, and extending over its whole extent. It would have added much to the pathological interest and value of this report, and the accompanying specimen, had the condition of the nervous ganglia in the abdomen been investigated, as a perversion of nutrition so extensive would, in all probability, involve as a proximate cause, some permanent modification of the ganglionic nervous system. Inflammatory disorganization of a semi-lunar ganglion being a well known cause of the corresponding lesion in the intestinal tract, it would be interesting to investigate the condition of these nervous centers as associated with a lesion so widely different in its character.

Of course, in the present state of science, our correspondent could not indulge in the faintest hope of even improving the condition of his patient, but while powerless to aid her individually, he has wisely endeavored to utilize the materials furnished by the case, for the advancement of science, and the possible benefit to future sufferers. We trust his good example will be followed by others. If gentlemen would report their failures, and furnish by means of autopsies the causes thereof, it might happen that one lost case thus given to the world would possess much greater value to science than many quasi successes which might be, after all, but cases of *post hoc non propter hoc.*

W. H.

---

***Placenta Prævia.***

Dr. Storer considered it of little advantage to temporize in these cases. Fortunately but few practitioners had many opportunities either of doing so or of testing in their own practice the different methods of treatment that had been suggested. He himself did not consider the old plan of forcibly delivering through the placenta nearly so good a one as that of Simpson, by detaching the placenta throughout its surface as rapidly as possible. Before relinquishing obstetric practice, he had been enabled to do this in more than one instance, and always with the happiest results.—*Gyn. Jour.*

